# Maternal Dietary Selenium Intake during Pregnancy and Neonatal Outcomes in the Norwegian Mother, Father, and Child Cohort Study

**DOI:** 10.3390/nu13041239

**Published:** 2021-04-09

**Authors:** Dominika Modzelewska, Pol Solé-Navais, Anne Lise Brantsæter, Christopher Flatley, Anders Elfvin, Helle Margrete Meltzer, Verena Sengpiel, Malin Barman, Bo Jacobsson

**Affiliations:** 1 Department of Obstetrics and Gynaecology, Sahlgrenska Academy, Institute of Clinical Science, Gothenburg University, SE-416 85 Gothenburg, Sweden; dominika.modzelewska@gu.se (D.M.); pol.sole.navais@gu.se (P.S.-N.); christopher.flatley@gu.se (C.F.); verena.sengpiel@obgyn.gu.se (V.S.); 2 Division of Infection Control, Environment and Health, Norwegian Institute of Public Health, N-0456 Oslo, Norway; annelise.brantsaeter@fhi.no (A.L.B.); HelleMargrete.Meltzer@fhi.no (H.M.M.); 3 Department of Pediatrics, The Queen Silvia Children’s Hospital, Sahlgrenska University Hospital, 416 50 Gothenburg, Sweden; anders.elfvin@vgregion.se; 4 Department of Obstetrics and Gynaecology, Sahlgrenska University Hospital/Östra, SE-416 85 Gothenburg, Sweden; 5 Department of Biology and Biological Engineering, Chalmers University of Technology, N-412 96 Gothenburg, Sweden; malin.barman@chalmers.se; 6 Institute of Environmental Medicine, Karolinska Institutet, SE-171 77 Stockholm, Sweden; 7 Department of Genetics and Bioinformatics, Domain of Health Data and Digitalisation, Institute of Public Health, N-0456 Oslo, Norway

**Keywords:** selenium, neonatal outcome, pregnancy, small for gestational age, Norwegian Mother, Father, and Child Cohort Study, MoBa, Medical Birth Registry of Norway, MBRN

## Abstract

Properly working antioxidant defence systems are important for fetal development. One of the nutrients with antioxidant activity is selenium. Increased maternal selenium intake has been associated with reduced risk for being small for gestational age and preterm delivery. Based on the Norwegian Mother, Father, and Child Cohort Study and the Medical Birth Registry of Norway, we investigated the association of maternal selenium intake from food and dietary supplements during the first half of pregnancy (*n* = 71,728 women) and selenium status in mid-pregnancy (*n* = 2628 women) with neonatal health, measured as two composite variables (neonatal morbidity/mortality and neonatal intervention). Low maternal dietary selenium intake (<30 µg/day) was associated with increased risk for neonatal morbidity/mortality (adjusted odds ratio (adjOR) 1.36, 95% confidence interval (95% CI) 1.08–1.69) and neonatal intervention (adjOR 1.16, 95% CI 1.01–1.34). Using continuous variables, there were no associations between maternal selenium intake (from diet or supplements) or whole-blood selenium concentration and neonatal outcome in the adjusted models. Our findings suggest that sufficient maternal dietary selenium intake is associated with neonatal outcome. Adhering to the dietary recommendations may help ensure an adequate supply of selenium for a healthy pregnancy and optimal fetal development.

## 1. Introduction

Maternal nutrition has been shown to be important for pregnancy and fetal development [1,2]. During pregnancy, the continuous physiological changes in the mother and the development of the fetus are dependent on the supply of specific nutrients [1,3,4]. Both nutrient deficiency and oversupply have been associated with adverse pregnancy, fetal, and neonatal outcomes, including preterm delivery, intrauterine growth restriction, and low birth weight, which are the leading causes of neonatal death [1,2]. Owing to complex and diverse biological processes, nutritional requirements and sensitivity to abnormal nutrient consumption change throughout pregnancy [3]. Understanding the significance of maternal nutrition can help establish dietary strategies to sustain pregnancy and support fetal maturation [5].

One of the essential nutrients is the trace element selenium. It plays a role in preventing cell damage through its antioxidant activity. Selenium, in the form of selenoproteins or antioxidant enzymes, regulates the distribution of reactive oxygen species or free radicals [6,7], thus preventing oxidative stress. A properly working antioxidant defence system is required for balancing naturally occurring oxygen tension at different gestational ages [3]. The lack of oxidative stress regulation has been associated with preeclampsia [8,9], preterm delivery [10], or small for gestational age (SGA) [11]. Additionally, neonatal health status has been shown to be dependent on the presence of free radicals [12]. The development of neonatal morbidities, such as perinatal hypoxic–ischaemic encephalopathy, retinopathy of prematurity, necrotizing enterocolitis, or bronchopulmonary dysplasia, has been shown to be related to oxygen free radicals [12]. 

Previously, our research group detected an association between a single nucleotide polymorphism in the gene responsible for incorporating selenoproteins (eukaryotic elongation factor selenocysteine tRNA-specific, *EFFSEC*) and gestational duration and preterm delivery (delivery <37 weeks of gestation, PTD), when using selenium at a continuous rate [13]. The importance of selenium for gestational duration was confirmed in our population-based epidemiological study [14]. Mothers who had higher dietary selenium intake during pregnancy had a longer-lasting pregnancy and a decreased risk for preterm delivery [14]. Similarly, in the same study population, higher dietary selenium intake was associated with increased birth weight and a reduced risk of SGA (birth weight for gestational age <10th percentile) [15]. 

Gestational age at delivery and birth weight are two important determinants of neonatal health. Being born too early (PTD) or with too small a birthweight (SGA) is strongly associated with neonatal mortality or morbidity [16]. Given that we have previously found maternal selenium intake during pregnancy to be associated with both SGA [15] and PTD [14], and that it is well know that SGA and PTD are associated with an increased risk for adverse neonatal outcomes, here, we hypothesized that there may be an association between maternal selenium intake and impaired neonatal health. 

The aim of this study is to examine whether self-reported maternal dietary and supplemental selenium intake during the first half of pregnancy, and maternal selenium status indicated by whole-blood selenium concentration at week 18, were associated with neonatal health assessed in the form of two composite variables: neonatal mortality/morbidity and neonatal intervention. 

## 2. Materials and Methods

### 2.1. Data

This study is based on the Norwegian Mother, Father, and Child Cohort Study (MoBa), which also includes data from the Medical Birth Registry of Norway (MBRN). The MBRN is a national health registry that contains information about pregnancy, delivery, and the health of the mother and neonate for nearly all births in Norway since 1967. Through compulsory notifications, the MBRN collects information about pregnancy, delivery, and the health of the mother and the neonate. It also contains data from neonatal and paediatric wards on congenital malformations, neonatal diagnoses, and procedures performed on infants transferred to those units [17]. 

MoBa is a population-based, ongoing, prospective pregnancy cohort study conducted by the Norwegian Institute of Public Health. Voluntarily participating women were recruited from throughout Norway between 1999 and 2008. Of all the invited women, 41% consented to participate [18]. The cohort now includes 114,500 children, 95,200 mothers, and 75,200 fathers. In MoBa, women filled in multiple questionnaires at different time points during pregnancy and after delivery. In this study, we used data obtained from two self-reported questionnaires: the baseline questionnaire (Q1) completed around gestational week 15 and a food frequency questionnaire (FFQ) completed around gestational week 22. The participants were asked to donate biological samples during pregnancy and at birth [18]. Blood samples were obtained from both parents during pregnancy and from mothers and children (umbilical cord) at birth. In this study, we were given access to data on whole-blood selenium concentration analyzed in a subgroup of participants sampled during the routine free ultrasound examination at gestational week 18. This study is based on version 10 of the quality-assured data files released for research in 2017.

The establishment of MoBa and the initial data collection was based on a license from the Norwegian Data Protection Agency and approval from the Regional Committees for Medical and Health Research Ethics. The MoBa cohort is now based on regulations related to the Norwegian Health Registry Act. Written informed consent was obtained from each participant. The Regional Committees for Medical and Health Research Ethics approved this study (2015/2425/REK South-East A).

### 2.2. Study Population

To be included in the current study, women needed to have had responded to the two first questionnaires (Q1 and FFQ) and to be registered in the MBRN with singleton live births without congenital malformations (*n* = 84,855; excluded, *n* = 29,384). We excluded pregnancies with gestation lasting <22 weeks and >42 completed weeks (*n* = 534), and those with invalid dietary reports (energy intake < 4.5 MJ, > 20 MJ, or > 3 blank pages) (*n* = 1493). Among women who participated in MoBa with more than one pregnancy, only their first enrolled pregnancy was included (excluded, *n* = 10,925). Finally, we excluded mother–child pairs with birth weight or selenium intake values that were >4 standard deviations from the mean (*n* = 175), resulting in a final study population of *n* = 71,728 for the analysis of selenium intake and *n* = 2628 for the analysis of selenium status. The study population used in this study was the same as that in our previous selenium study on birth weight and SGA status [15]. 

### 2.3. Maternal Selenium Intake from Food and Dietary Supplements

Maternal daily selenium intake was estimated using the information obtained from a semiquantitative FFQ developed and validated for use in MoBa [19,20]. The FFQ was designed to capture dietary habits and intake of dietary supplements during the first 4–5 months of pregnancy. The MoBa FFQ was used from March 2002 through the remaining recruitment period. Briefly, women were asked to report their average intake of 255 different foods and dishes since the beginning of their pregnancy. The frequency intervals ranged from never to several times daily. The FFQ was validated using a 4-day food record and biological markers as reference methods. The results showed that the MoBa FFQ produces a realistic and relatively precise estimate of the habitual intake of energy, nutrients, and food groups in pregnant Norwegian women, and is a valid tool for categorizing pregnant women according to high and low intakes of energy, nutrients, and foods [19,20,21,22].

We converted consumption frequencies into food amounts, and calculated the daily intake of energy and nutrients using FoodCalc [23] and the Norwegian food composition table [24]. On the last page of the FFQ, the participants were asked to report the use of dietary supplements by writing the names, brand, intake frequency, and amount [14]. To calculate the selenium amount contributed by the dietary supplements, we used a database of dietary supplements [25] and manufacturer-declared nutrient content information. The selenium from supplements was differentiated into inorganic (selenite and selenate) and organic (selenomethionine and selenized yeast) forms. Overall, we considered three sources of selenium: selenium originating from diet, organic selenium from supplements, and inorganic selenium from supplements.

### 2.4. Selenium Status

Maternal blood samples donated in gestational week 18 were shipped via overnight mail in a vacutainer for long-term freezing in the MoBa biobank repository [26]. Maternal whole-blood samples from 3000 individuals were retrieved from the biobank in 2015 for the Human Environmental Biobank project (MoBa Etox) at the Norwegian Institute of Public Health [27]. Selenium analysis was performed in 2015 at Lund University (Sweden) using an inductively coupled plasma mass spectrometer (IiCAP Q; Thermo Fisher Scientific (Bremen) GmbH) equipped with a collision cell having kinetic energy discrimination and helium as the collision gas. The detection limit was 3.2 μg/L, and the coefficient of variation was 1.5%. All samples were above the detection limit. The analytical accuracy was verified against a certified reference material (Seronorm Trace Elements Whole Blood L-1 and L-2; SERO AS, Billingstad, Norway). Blood sample collection and selenium detection followed previously detailed procedures [14,15,27].

### 2.5. Neonatal Outcomes

Data on neonatal outcomes were extracted from the MBRN. Two composite variables for neonatal outcomes were created: neonatal mortality/morbidity and neonatal intervention.

In terms of neonatal mortality and morbidity outcomes, we included children who died within 28 days after birth, with 5 min Apgar score < 4, or with any of the following International Classification of Disease (10th edition) codes [28]: birth asphyxia (P21), chronic respiratory disease originating in the perinatal period (P27), intracranial (non-traumatic) hemorrhage of the fetus and newborn (P52), meconium ileus/necrotizing enterocolitis (P75, P76, P77, P78.0, P78.1), other disturbances in the cerebral status of newborns (P91.0, P91.1, P91.2, P91.6), retinopathy of prematurity (H35.1), bacterial sepsis in the newborn (P36), or other infections specific to the perinatal period (P39). 

Neonatal intervention included transfer to the neonatal intensive care unit (NICU), ventilator or continuous positive airway pressure treatment, or treatment with systemic antibiotics.

### 2.6. Statistical Analyses

We used logistic regression to assess the relationship between different selenium exposures and neonatal outcomes. Results are provided from both unadjusted and adjusted models. Potential confounders were obtained from three sources: Q1 (pre-pregnancy maternal weight, maternal height, maternal education, and smoking during pregnancy), FFQ (nausea at the time of filling in the FFQ, alcohol consumption, dietary fiber intake, iodine intake, protein intake, and total energy), and MBRN (maternal age at delivery, and parity). Maternal weight and height were considered in the form of body mass index (BMI) according to the World Health Organization classification: <18.5, 18.5–24.9, 25.0–29.9, or >30.0 kg/m^2^. Parity was categorized as the number of previous births: 0, 1, 2, or ≥3. Maternal education was recorded in three strata: <13, 13–16, and >16 years. Smoking was classified as non-smoking, occasional, or daily smoking. Alcohol consumption, marital status, nausea, folic acid supplementation, and planned pregnancy were assessed as binary variables (yes or no). Iodine intake was ranked in quintiles. Dietary fiber, total protein, total energy intake in kJ, and maternal age at delivery were added as continuous variables. Women with missing information were removed from the adjusted regression analyses (66,908 women (93%) had information on all covariates used in the main analyses).

Four different selenium exposures were used: selenium intake from diet, inorganic selenium intake from supplements, organic selenium intake from supplements, and selenium blood concentration. We conducted the analyses of selenium on a continuous and dichotomous scale. We standardized (subtracted the mean and divided by the standard deviation) continuous maternal selenium intake and selenium whole-blood concentration to determine the risk for neonatal outcomes with increased selenium intake by 1 standard deviation, which was 14.3 µg/day for dietary selenium intake, 10.4 µg/day for inorganic selenium from supplements, 33 µg/day for organic selenium from supplements, and 23.4 µg/L for whole-blood selenium concentration. We further assessed the risk of neonatal outcomes with respect to total daily selenium intake (selenium from diet and supplements) <30 and >120 μg. The lower and higher cut-offs are half and double the recommended daily selenium intake, respectively. In order to assess whether the association between dichotomized maternal selenium intake and neonatal outcome is mediated by SGA/PTD, we also estimated the association of total daily maternal selenium intake < 30 µg and SGA status with PTD using logistic regression adjusted for the same set of covariates as the models for neonatal outcomes. SGA status was defined as birth weight <10th percentile based on Skajerven’s gestational age-based growth curves [29]. PTD was defined as delivery <37th week of gestation. IBM SPSS Statistics version 27.0 and R version 3.5.0 were used to perform the statistical analyses.

## 3. Results

### 3.1. Study Population

The selection criteria used in this study resulted in a total of 71,728 mother–child pairs (84.5% of the 84,855 eligible mothers, i.e., those who had responded to Q1 and FFQ and were registered in the MBRN with singleton live births) available for the analyses of maternal dietary selenium intake, and 2628 for the analyses of maternal blood selenium concentration.

Most of the mothers were between 25 and 34 years of age (76%), had >12 years of education (66%), had a BMI between 18.5 and 29.9 kg/m^2^ (85%), were never active smokers (91%) or never passive smokers (88%), and did not drink alcohol (89%) (Table 1). Half of the women were nulliparous (53%). Nausea in the second trimester was reported by 11% of women (Table 1).

Higher selenium intake was associated with higher age, education, and parity (Kruskal–Wallis test, *p* < 0.001) (Table 1).

### 3.2. Selenium Intake

The median selenium intake from diet was 53 μg/day (25th–75th percentile: 44–62 μg/day). Of the whole study population, 29% met the Nordic recommendation for selenium for pregnant women of 60 μg/day [30] through dietary intake. Selenium dietary intake mainly originated from cereals and grains (on average, 41% of total selenium intake), fish and seafood (29%), meat (23%), and egg and dairy products (20%).

Approximately 33% of women (*n* = 23,336) reported using selenium-containing dietary supplements. Of these, 28% took selenium supplementation only in the organic form, around 4% only in the non-organic form, and 1% in both forms. Women obtaining selenium from supplements did not differ from women not taking supplements in terms of their mean dietary selenium intake (54 μg/day in both groups). Supplementation resulted in a substantially higher total selenium intake (Table 1). The median total selenium intake (from diet and supplements) was 102 μg (83–123 μg) (Table 1). Of women taking selenium-containing supplements, 94% met the recommended intake of >60 μg/day [30].

### 3.3. Blood Selenium Concentration

**Figure 1:**
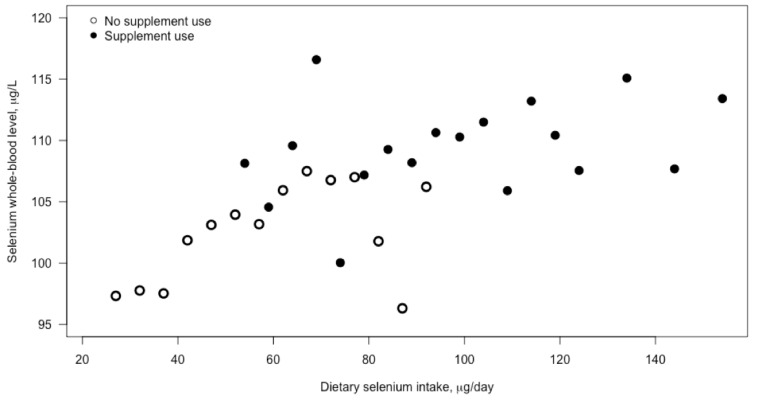
Scatterplot of selenium concentration in blood and dietary selenium intake. The figure shows the mean selenium concentration in blood in different groups of women according to their selenium intake. Selenium intake (μg/day) was categorized into intervals: 23–27, 28–32, 33–37 up to 120–124, 125–144, and 145–154+. The relationship between intake and blood concentrations is depicted separately for women who reported using selenium-containing dietary supplements (black dots) and women who reported no use of such supplements (open dots). Selenium intake among non-supplement users originated from diet, whereas intake among supplement users is presented as the sum of selenium from diet and supplements.

The median selenium blood concentration was 105 μg/L (25th–75th percentile: 89–117 μg/L). Selenium blood concentration was weakly associated with selenium intake (correlation = 0.11, *p* < 0.001). Significantly higher blood concentrations of selenium were found among women taking selenium-containing supplements (110 μg/L, 25th–75th percentile: 87–115 μg/L) than in women not taking supplements (102 μg/L, 25th–75th percentile: 94–121 μg/L) (*t*-test (degrees of freedom): *t*(46,225), *p* < 0.001) (Figure 1).

### 3.4. Neonatal Outcomes

In terms of neonatal morbidity, the most common conditions were birth asphyxia (3.3%) and bacterial sepsis in the newborn (1.3%) (Table 2). A total of 3462 children (4.8%) had at least one of the selected morbidities or died within 28 days of birth. With respect to neonatal intervention, NICU admission (15.2%) or the use of systemic antibiotics (2.8%) were the most common (Table 2). A total of 10,973 children (15.3%) had at least one intervention.

### 3.5. Selenium Exposure and Neonatal Outcomes

**Figure 2:**
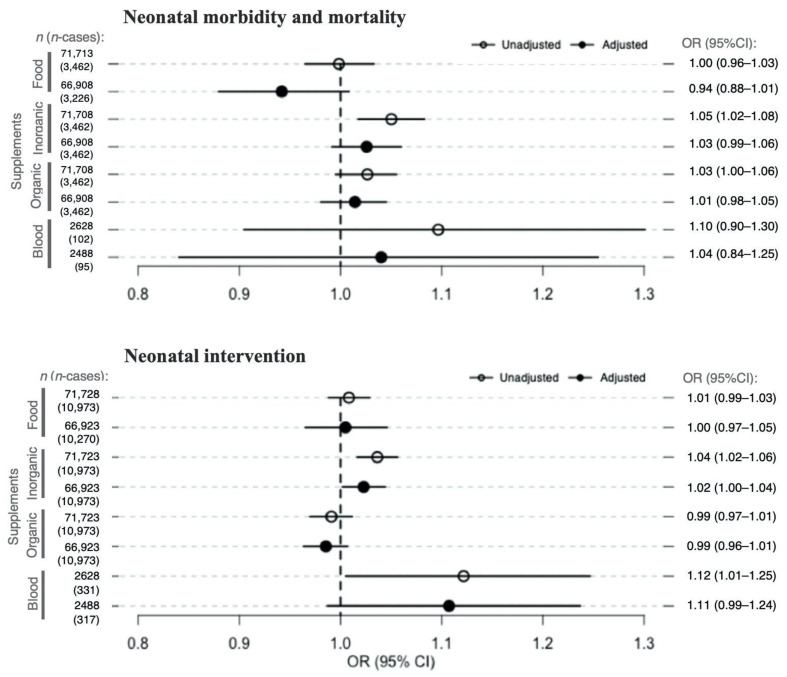
Associations between maternal selenium intake from diet, selenium intake from dietary supplements, and blood selenium concentration (all continuous) and neonatal outcomes. The figure presents the ORs and 95% CIs for neonatal outcomes, neonatal mortality/morbidity (top plot) and neonatal intervention (bottom plot), per selenium exposure divided as follows: selenium from diet intake—1 standard deviation of dietary selenium intake (14.3 μg/day), inorganic supplements—1 standard deviation of inorganic selenium intake from supplements (10.4 μg/day), organic supplements—1 standard deviation of organic selenium intake from supplements (33.0 μg/day), and 1 standard deviation of selenium whole-blood concentration (23.4 µg/L). Circles and full dots represent the ORs for the unadjusted and adjusted models, respectively. Specific ORs and 95% CIs are depicted in the right panel of the figure. Models with maternal selenium intake from diet or supplementation as exposure were adjusted for maternal age at delivery, maternal pre-pregnancy body mass index (BMI), parity, maternal smoking during pregnancy, passive smoking, nausea during the second trimester, maternal education, fiber intake, iodine intake, protein intake, n-3 intake from diet, and total energy intake. Models were also mutually adjusted for the different selenium sources. Models with maternal selenium concentration in whole blood as exposure were adjusted for maternal age at delivery, maternal pre-pregnancy BMI, parity, maternal smoking during pregnancy, passive smoking, and maternal education. The sample sizes on which the analyses were run are represented by the *n* value in the left panel of the figure. Abbreviations: *n*, sample size; n-cases, number of cases in each sample; OR, odds ratio; CI, confidence interval.

A statistically significant association between selenium derived from supplements and the composite neonatal outcomes was found only in the three unadjusted models: inorganic selenium supplements and neonatal mortality and morbidity (OR 1.05 per 10.4 μg/day, 95% CI 1.02–1.08), inorganic selenium supplements and neonatal intervention (OR 1.04 per 10.4 μg/day, 95% CI 1.02–1.06), and selenium blood concentration and neonatal intervention (OR 1.12 per 23.4 µg/L selenium concentration in whole blood, 95% CI 1.01–1.25) (Figure 2). The associations were no longer significant after adjusting for potential confounders (Figure 2).

**Figure 3:**
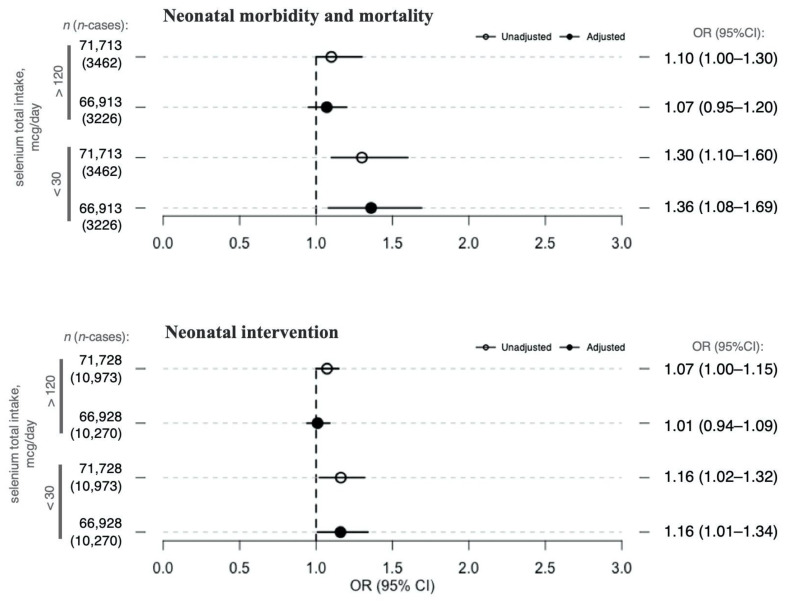
Associations between maternal total selenium intake (dichotomized) and neonatal outcomes. The figure presents the ORs and 95% CIs for neonatal outcomes, neonatal mortality/morbidity (top plot) and neonatal intervention (bottom plot), per selenium exposure divided as follows: selenium intake < 30 μg/day (compared with selenium intake ≥ 30 μg/day) or selenium intake > 120 μg/day (compared with selenium intake ≤ 120 μg/day). Lower selenium intake (<30 μg/day) is attributable to selenium intake from diet only, whereas higher selenium intake (>120 μg/day) is attributable to selenium intake from both diet and supplements. Circles and full dots represent ORs for the unadjusted and adjusted models, respectively. Specific ORs and 95% CIs are depicted in the right panel of the figure. Models were adjusted for maternal age at delivery, maternal pre-pregnancy body mass index (BMI), parity, maternal smoking during pregnancy, passive smoking, nausea during the second trimester, maternal education, fiber intake, iodine intake, protein intake, n-3 intake from diet, and total energy intake. Sample sizes on which the analyses were run are represented by the *n* value in the left panel of the figure. Abbreviations: *n*, sample size; *n*-cases, number of cases in each sample; OR, odds ratio; CI, confidence interval.

Total selenium intake < 30 µg/day (2% of all women, observed only among non-supplement users; Table 3) compared with selenium intake ≥ 30 µg/day was significantly associated with a higher risk for neonatal mortality/morbidity, in both unadjusted and adjusted models (adjOR 1.36, 95% CI 1.08–1.69) (Figure 3). After adjustment, total selenium intake < 30 µg/day was also associated with neonatal intervention (adjOR 1.16, 95% CI 1.01–1.34) (Figure 3). Total selenium intake < 30 µg/day was not associated with SGA (adjOR 1.14, 95% CI 0.96–1.35) or PTD (adjOR 1.14 95% CI 0.90–1.42) in the adjusted models. Total selenium intake > 120 µg/day (9% of all women, observed only among supplementing women; Table 3) compared with selenium intake ≤ 120 µg/day was not associated with any of the neonatal outcomes in either unadjusted or adjusted models (Figure 3).

## 4. Discussion

The main finding of this study is an increased risk for at least one of the specified neonatal interventions or neonatal morbidities, or neonatal death, for babies whose mothers reported selenium intake < 30 μg/day. No association was found between selenium intake (from diet or supplements, when analyzed as continuous variables), total selenium intake > 120 μg/day or whole-blood selenium concentration and neonatal mortality/morbidity or neonatal intervention.

Low maternal serum selenium levels affect maternal antioxidant status, which, in turn, contributes to the risk of oxidative stress in the neonate [31]. Oxidative stress is associated with neonatal morbidities, including retinopathy of prematurity, persistent ductus arteriosus, necrotizing enterocolitis, intracranial hemorrhage, and hypoxic–ischaemic encephalopathy [31]. In this study, maternal total selenium intake < 30 μg/day was associated with the neonatal composite outcome variables. As with all nutrients, the selenium status of the fetus is dependent on placental nutrient flow. The placental transfer of selenium is limited, and the selenium concentration found in umbilical cord blood is approximately 65% of the maternal serum concentration [31,32]. Preterm born infants tend to have lower serum selenium concentrations in comparison to term infants [31].

**Figure 4:**
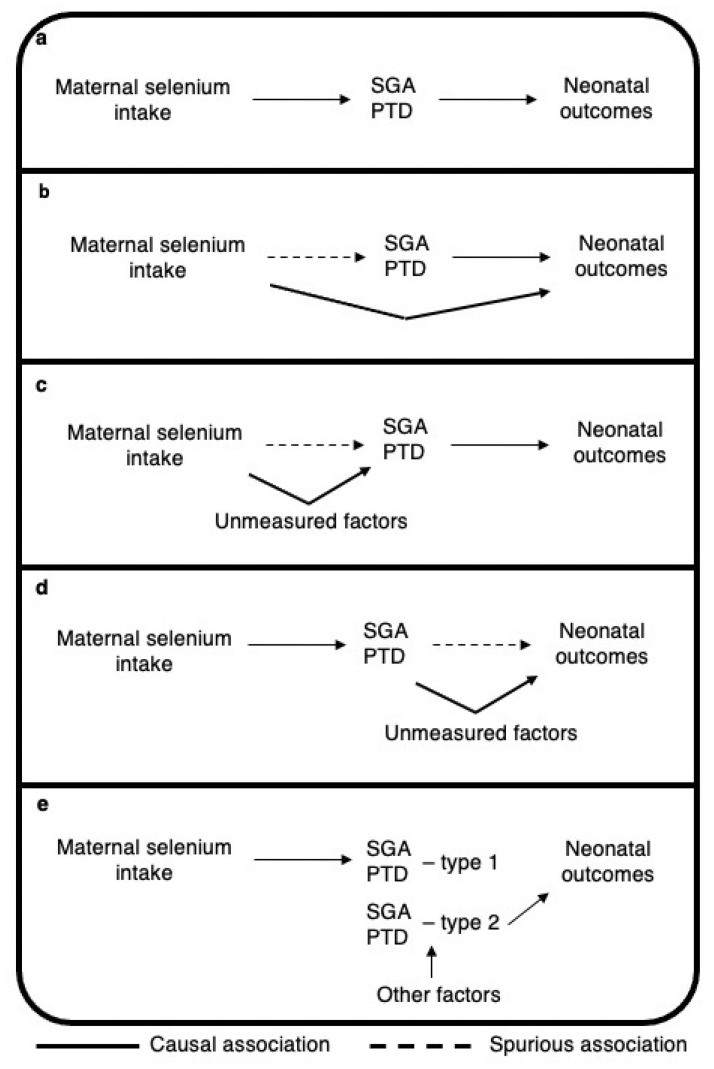
Possible causal structure of the association between maternal selenium intake, SGA, and neonatal outcomes. (**a**) selenium intake effect fully mediated; (**b**) selenium intake partially mediated; residual confounding creating spurious association between selenium intake and SGA/PTD (**c**) or SGA/PTD and neonatal outcomes (**d**); (**e**) different types of SGA/PTD being differently associated with maternal selenium intake and neonatal outcomes. The figure shows four directed acyclic graphs displaying the possible associations between maternal selenium intake, SGA, and neonatal outcomes. Abbreviation: SGA, small for gestational age; PTD, preterm delivery.

In one of our previous studies based on the MoBa cohort, we found a protective effect of increased maternal dietary selenium intake on SGA (OR 0.92 per 14.3 μg/day selenium) [15]. In another study in MoBa we found that SGA was associated with mortality/morbidity or neonatal intervention (OR > 3 for both outcomes) [33]. Similarly, we have previously found increased maternal dietary intake to be associated with a decreased risk of PTD (hazard ratio (HR) 0.88 per 14.6 μg/day) [14]. PTD is a known risk factor for neonatal mortality and morbidity [34]. Therefore, we expected to observe a decreased risk of adverse neonatal outcomes with increased maternal selenium intake, either fully (Figure 4a) or partly mediated by SGA/PTD (Figure 4b), or with residual confounding (Figure 4c or Figure 4d). However, we did not find any association between increased maternal selenium intake (continuous variable) and adverse neonatal outcomes in the current study. We hypothesize that this lack of association may be due to different types of SGA/PTD being differently associated with maternal selenium intake and neonatal outcomes (Figure 4e). Maternal selenium intake might contribute to normal fetal growth and prevent selenium-related types of SGA/PTD, which may not be associated with the adverse neonatal outcomes studied (Figure 4e). Conversely, babies born SGA or preterm owing to reasons other than selenium-related pathological processes might perform worse and have neonatal morbidities (Figure 4e). As in our previous paper on maternal caffeine consumption and neonatal outcomes [33], our findings in the current study might support the hypothesis that babies born SGA or preterm because of different causes might differ in neonatal health status. Similarly, Hernandez-Diaz proposed an explanation for the higher death risk in SGA neonates born to non-smoking mothers than in those born to smoking mothers. They suggested that factors affecting fetal growth and SGA among non-smoking mothers might be more aggressive than smoking itself, and lead to more serious health complications [35].

In this study, there was no direct association between higher (>120 μg/day) maternal total selenium intake and adverse neonatal outcomes. This lack of association might be due to most of the women having an adequate selenium intake, close to the Nordic recommendations of 60 ug/day. This level of intake ensures adequate saturation of selenium-dependant enzymes [36]. Only approximately 6434 (9%) women had daily selenium intake > 120 μg, and only 139 of them had an intake of > 400 μg/day. Furthermore, higher intake levels were observed only among women taking supplements.

The major strength of this study is that the analysis is based on a comprehensive dataset. Adverse neonatal outcomes are rare, which implies that large datasets are needed. With a sample size of 71,728 mother–child pairs, this study is the largest study examining the effect of selenium intake from diet and supplements on whole-blood selenium concentration and neonatal outcomes. However, we must note that the effects observed are clinically relevant on the population level, but may not be critical on an individual basis. As the study included mothers from throughout Norway, the analysis was performed on mothers who differ in multiple characteristics, such as different areas and regions of residence, socioeconomic classes, or dietary habits. Detailed questionnaire data and the MBRN linkage helped us control for major maternal characteristics. The FFQ allowed the estimation of maternal selenium intake based on a broad set of food items and supplements. Neonatal outcomes were retrieved from the national registry (MBRN), guaranteeing that registration was performed by qualified health-care professionals.

The limitations of this study were related to the observational study design. Although we had information on the major maternal characteristics, we cannot rule out the possibility that residual or unmeasured confounders exist. Selenium as part of nutrition is effective, but the effect might be modified by interactions with other nutrients. We acknowledge that other confounders, such as choline or other nutrients (among others, zinc, vitamins, or heavy metals), could affect the observed results. However, we adjusted for the lifestyle factors that can be considered as proxies for several of these confounders.

In addition, possible self-selection biases might be present. MoBa includes only voluntarily participating women who differ from the general population of women giving birth in Norway in some exposure and outcome characteristics; for example, the participants are older, better educated, and include fewer smokers. However, a comparison of eight exposure–outcome associations reported in both MoBa and the MBRN showed no differences in the selected associations [37]. In addition, the subgroup of women with available information on whole-blood selenium concentration differs from the MoBa cohort. More women in this study were non-smokers and had higher education levels than women in the entire MoBa [27].

Another limitation was that the exposure and outcome variables may be imprecise. Selenium content varies in plants due to high variety in soil selenium concentrations and availability, and in meat or dairy products, depending on selenium concentrations in the fodder, making selenium intake estimation difficult. Additionally, selenium was estimated using self-reported dietary habits, which are prone to bias. With respect to neonatal outcomes, the MBRN collects information from maternity units; thus, diagnoses made after the babies were released from the hospital are missed.

## 5. Conclusions

Low maternal selenium intake might contribute to adverse neonatal outcomes. Adhering to the dietary recommendations and eating a varied diet will help to ensure an adequate supply of selenium for a healthy pregnancy and optimal fetal development. We previously found an association between maternal selenium intake at a continuous rate and risks for both SGA and PTD, known to be associated with adverse neonatal outcome. However, in the present study no association was found between continuous selenium intake or whole-blood selenium concentration and adverse neonatal outcomes. We suggest that different etiologies of SGA/PTD will lead to different neonatal outcomes; SGA/PTD associated with maternal selenium intake will have milder adverse neonatal outcomes than SGA/PTD caused by other, more severe factors.

## Figures and Tables

**Table 1:** Dietary intake and total (diet and supplements) selenium intake by maternal characteristics.

				Dietary Selenium Intake, μg/day		Total Selenium Intake, μg/L	
Characteristics		*n*	%	Median	25th–75th Percentile	Median	25th–75th Percentile
Total population		71,728	100	53	44–62	102	83–123
Maternal age, years	<25	8239	11	50	41–61	101	83–125
	25–29	24,313	34	52	43–62	101	83–122
	30–34	30,410	42	53	45–63	102	84–123
	>34	8766	12	54	46–64	104	85–125
Maternal education, years	<12	22,286	31	51	42–62	102	84–125
	12–16	29,757	41	53	44–62	101	83–123
	>16	18,150	25	54	46–64	102	83–122
	Missing	1535	2	51	42–62	99	82–122
Pre-pregnancy BMI, kg/m^2^	<18.5	2130	3	53	44–64	103	83–126
	18.5–24.9	45,976	64	53	45–63	102	83–123
	25–29.9	15,134	21	52	43–61	101	84–123
	>30	6633	9	51	42–61	102	84–122
	Missing	1855	3	52	44–62	99	81–121
Parity	0 *	38,169	53	52	43–62	102	84–123
	1	21,557	30	53	44–62	101	83–122
	2	9652	13	54	45–63	101	83–121
	≥3	2293	3	54	45–64	104	82–128
	Missing	57	1	56	45–63	102	83–115
Smoking during pregnancy	Never	65,504	91	51	42–62	105	83–127
	Occasionally	3876	5	53	44–62	102	83–123
	Daily	1943	3	52	43–63	102	84–123
	Missing	405	1	52	43–62	99	79–121
Passive smoking	No	62,767	88	53	44–62	102	83–123
	Yes	7580	11	52	43–63	103	83–126
	Missing	1381	2	51	43–63	102	83–126
Alcohol consumption	No	63,806	89	53	44–62	102	84–123
	Yes	7922	11	54	46–63	101	82–122
Nausea during the second trimester	No	63,504	89	53	44–62	102	84–123
	Yes	8224	11	52	42–62	100	81–122
Iodine intake, μg/day	<89	17,637	25	43	36–50	91	74–112
	89–121	17,564	24	50	43–58	98	81–118
	121–162	18,354	26	55	48–63	104	87–124
	> 162	18,094	25	63	55–73	114	95–135
Protein intake, g/day	<76	23,952	33	42	36–48	89	72–108
	76–93	23,826	33	65	58–74	114	97–136
	>93	23,950	33	53	47–59	101	85–121
Total energy intake, kJ/day	<8392	23,950	33	44	37–50	91	75–112
	8392–10,460	23,832	33	63	55–73	112	94–134
	>10,460	23,946	33	53	46–60	102	85–121
Gestational age at birth, weeks + days	≤27 + 6	126	0	53	47–62	114	83–133
	28 + 0 to 31 + 6	306	0	50	41–60	98	85–114
	32 + 0 to 36 + 6	2836	4	53	44–62	103	86–123
	≥37 + 0	67,827	95	53	44–62	102	83–123
	Missing	594	1	53	44–62	100	81–120
Birth weight, g	<1500	398	1	52	44–62	101	82–121
	1500–2500	1751	2	52	43–61	102	86–122
	>2500	69551	97	53	44–62	102	83–123
	Missing	31	0	52	47–58	107	88–134

The table shows the median daily total selenium intake (from diet and supplements) and selenium intake from diet according to maternal characteristics in the Norwegian Mother, Father, and Child Cohort Study (*n* = 71,728). Selenium intake was estimated using a food frequency questionnaire completed by women at gestational week 22. * Parity refers to the number of births before the current pregnancy (equal to zero means that the woman had no previous children, primiparous). Abbreviations: BMI, body mass index; *n*, sample size.

**Table 2:** Number of children included in groups of morbidity or neonatal death and neonatal intervention.

	Morbidity (ICD-10)	Number of Children	Percentage
Total		71,728	100
Neonatal mortality and morbidity	Apgar score < 4 at 5 min	137	0.19
	Birth asphyxia (P21)	2416	3.3
	Other disturbances in the cerebral status of the newborn (P910–916)	30	0.04
	Intracranial (non-traumatic) hemorrhage of the fetus and newborn	154	0.21
	Retinopathy of prematurity (H351)	22	0.03
	Meconium ileus/necrotizing enterocolitis (P75, P76, P77, P780, P781)	31	0.04
	Chronic respiratory disease originating in the perinatal period (P27)	77	0.11
	Bacterial sepsis in the newborn (P36)	906	1.3
	Other infections specific to the perinatal period (P39)	252	0.35
	Neonatal death	629	0.88
Total neonatal mortality and morbidity		3462	4.83
Neonatal intervention	Systemic antibiotics	1976	2.8
	Ventilator	363	0.51
	CPAP	935	1.30
	NICU admission	10,942	15.25
Total neonatal intervention		10,973	15.30

The table shows the number and percentage of children who died within 28 days after birth or were affected by a specific morbidity or intervention in the Norwegian Mother, Father, and Child Cohort Study (*n* = 71,728). The totals of neonatal mortality and morbidity or neonatal intervention represent the number of children having at least one diagnosis/intervention. Therefore, the total number is lower than the sum of all diagnoses/interventions. Abbreviations: ICD-10, International Statistical Classification of Diseases and Related Health Problems, 10th edition; CPAP, continuous positive airway pressure; NICU, neonatal intensive care unit.

**Table 3:** Maternal selenium intake from diet and supplements and maternal blood selenium concentration, with respect to neonatal outcomes.

		Total, *n* = 71,728		Neonatal Mortality/Morbidity				Neonatal Intervention			
				No		Yes		No		Yes	
		Median 25th–75th Percentile		Median 25th–75th Percentile		Median 25th–75th Percentile		Median 25th–75th Percentile		Median 25th–75th Percentile	
Dietary selenium intake, μg/day		53	44–62	53	44–62	53	44–62	53	44–62	53	44–62
Inorganic selenium supplements, μg/day		50	35–75	50	35–75	50	36–75	50	35–75	50	36–75
Organic selenium supplements, μg/day		30	21–43	30	21–43	30	25–50	30	21–43	30	21–36
Selenium blood concentration, μg/L		102	89–117	102	89–117	106	89–121	102	89–117	105	92–119
		*n*	**%**	*n*	**%**	*n*	**%**	*n*	**%**	*n*	**%**
Total selenium (diet and supplements), μg/day	<30	1180	2	1101	2	79	2	976	2	204	2
	30–120	64,114	89	61,064	89	3036	88	54,383	90	9731	89
	>120	6434	9	6086	9	347	10	5396	8	1038	9

The median daily selenium intake from diet, selenium intake from inorganic or organic supplements, and maternal selenium concentrations in whole blood according to neonatal outcomes in the Norwegian Mother, Father, and Child Cohort Study (*n* = 71,728) are shown. The table shows the number and percentage of women with daily selenium intake < 30 μg, between 30 and 120 μg, and >120 μg with respect to neonatal outcomes. The reported *p*-values show the significance of the two-tailed *t*-test.

## Data Availability

The consent provided by the participants is not open for storage of data on an individual level in repositories or journals. Researchers who want access to data sets for replication should submit an application to datatilgang@fhi.no. Access to data sets requires approval from the Regional Committee for Medical and Health Research Ethics in Norway and an agreement with MoBa.
